# Epigenetic Trajectories of Aging and Reprogramming

**DOI:** 10.1093/geroni/igab046.2508

**Published:** 2021-12-17

**Authors:** Peter Niimi, Morgan Levine, and Margarita Meer

**Affiliations:** Yale University, New Haven, Connecticut, United States

## Abstract

The epigenetic landscape is remodeled with age, bringing about widespread consequences for cell function. With the revolutionary discoveries by Yamanaka and Takahashi, as well as those that built on this work, the transcription factors Oct4, Sox2, KLF4, and C-Myc (OSKM) can be expressed in a variety of cells, including fibroblasts, to make iPSCs. Once cells are reprogrammed, they show an erasure of epigenetic remodeling, suggesting an avenue to reverse aging. It has been recently shown that ectopic expression of three factors, OSK, can restore vision in mouse glaucoma model and reduces epigenetic age. It is not known the path epigenetic remodeling takes or whether all three factors, OSK, are required to remodel the epigenetic landscape. We hypothesize that during reprogramming, cells will reverse along a similar path they took during aging and eventually reverse along that path they took during differentiation. Alternatively, it may also be possible that cells take entirely new paths to reach a state of partial reprogramming or pluripotency. We used DNA methylation and RNA-seq as a multi-omics approach to map the trajectories cells make during aging, differentiation, and reprogramming. In human fibroblasts and hepatocytes, we tested the three-factor OSK mix, as well as pairwise factors OS, OK, and SK and individual Oct4, Sox2, and KLF4 for their effect on cell trajectories. This study provides a dynamic model for epigenetic changes in aging, differentiation, and reprogramming and highlights barriers and bottlenecks throughout the process.

